# An ontological framework for the formalization, organization and usage of TCM-Knowledge

**DOI:** 10.1186/s12911-019-0760-9

**Published:** 2019-04-09

**Authors:** Hai Long, Yan Zhu, Lirong Jia, Bo Gao, Jing Liu, Lihong Liu, Heinrich Herre

**Affiliations:** 1 Institute of Medical Informatics, Statistics and Epidemiology (IMISE) University of Leipzig, Leipzig, Germany; 2 Institute of Information on Traditional Chinese Medicine, China Academy of Chinese Medical Sciences, Beijing, China

**Keywords:** Ontology, TCM, TCMLS, GFO, Semantic retrieval, TCM clinical decision support

## Abstract

**Background:**

The traditional Chinese Medicine Language System (TCMLS) is a large-scale terminology system, developed from 2002 on by the Institute of Information of Traditional Chinese Medicine (IITCM). Until now, more than 120,000 concepts, 300,000 terms and 1.27 million semantic relational links are included. Its top-level framework, called TCMLS-semantic network (SN), provides an important basis for the standardization and mapping of traditional Chinese Medicine (TCM) terminology systems. Though, many data produced and stored in TCMLS have poor quality for historical reasons or because of human factors. There is a large number of classification errors or inconsistent expressions of terms remained in the current TCMLS- SN, which hamper an efficient utilization of the data stored in TCMLS in practical applications.

**Methods:**

We start with analyzing the technical specification based on TCMLS, considering some obvious classification errors and problems of ambiguity of semantic expressions in TCMLS-SN, followed with using a top-down approach for building a middle level ontology which is based on the framework General Formal Ontology (GFO), take into account the compatibility with TCM related concepts, turn out the results of a modification of the current TCMLS-SN, called GFO-TCM.

**Results:**

Through comparison with TCMLS-SN, according to viewpoints of GFO, some semantic types and relations were reconstructed within GFO-TCM. We propose a middle level ontology for TCMLS which may support entailment and ensure coherence, we also draw out a mapping which possess a more reasonable framework with a unified semantic criterion, it is application scenarios oriented and can be further updated and extended.

**Conclusions:**

The goal is to construct a formal middle-level ontology that is compatible with both the traditional medical terminology system and modern medical terminology standards. it is intended to satisfy functional requirements which are relevant for natural language processing, information extraction, semantic retrieval, clinical decision support in the field of traditional Chinese medicine. It also provides a foundation and methodology for building a large-scale, unified semantic and extensible knowledge graph platform.

## Background

The medical terminology system, as an effective means to establish a standardization of medical terms, plays a fundamental role in practical applications, including medical literature retrieval, clinical decision support, data mining and knowledge discovery. Ontology is a new technology for constructing medical terminology and knowledge systems. Compared with traditional methods, such as subject heading and taxonomy, ontology has various advantages: it has a logical foundation, it supports deductive reasoning and semantic retrieval, it has good extendibility and easy maintenance [1]. Ontology-based semantic networks provide common structured representation of knowledge. Its basic idea is to represent domain knowledge in the form of labeled graphs, whose nodes represent entities or concepts, whereas edges represent semantic relationships between these concepts [2]. The development of the TCM theory has been deeply influenced by the Chinese traditional philosophies in the past centuries, it was provided with rich oriental cultural connotation. After thousands of years of development and evolution, various regional cultural differences and modes of usage provided richness and diversity to its terminology system. Due to the lack of unified semantic specifications and standards, there is a large number of semantic ambiguity problems in the TCM literature, occurring in the phenomena of homonyms and synonyms [3–5]. These problems hinder the modernization of Chinese medicine and the application of information technologies, and also result in difficulties to promote and popularize international standards for TCM terminology. Moreover, there is a huge difference between the TCM terminology system and the Western medical terminology systems; both have different thinking modes and are based on divergent cognition principles [6–8]. This situation leads to great challenges for effective understanding, dialogue and integration between the two. In recent years, the TCM research field has paid much attention to basic research, obtaining relevant achievements in this, but in practical applications there are still a series of open problems. Considering this situation, the Institute of Information on Traditional Chinese Medicine (IITCM), an affiliation of the China Academy of Chinese Medical Sciences (CACMS), jointly worked with more than 10 Chinese medicine research institutes and universities in China, and established a language system of traditional Chinese medicine (TCMLS) through the joint efforts of more than 300 experts over 10 years since 2002. The TCMLS (originally called UTCMLS) draws on construction principles and the basic architecture of Unified Medical Language System (UMLS) [9], which consists of a “semantic network” and a “basic thesaurus”. The semantic network framework constitutes the architecture’s top level of TCMLS, which defines the most basic semantic types and semantic relationships in the field of TCM. In summary, the semantic type system provides a classification framework for TCMLS and the semantic relation system connects the concepts of TCM and forms a large complex semantic network. After more than 10 years of application and improvement, the technical specification based on TCMLS, “ISO / TS17938 Health information - Semantic network framework of traditional Chinese Medicine Language System”, was accepted and released by the International Organization for Standardization (ISO) in 2018 [10].

### Complexity and difficulties of constructing a top-level ontology in TCM

The construction of a domain top level ontology is a difficult task because core classes and principal relationships, being relevant in the domain, must be selected and specified. These classes and relationships are the basis for the establishment of sub domain ontologies and the development of related applications. The construction of a Top-level ontology for the TCM domain must take into consideration the subsequently discussed aspects.

### Complexity of TCM

TCM is closely related to the traditional Chinese culture. On the one hand, ancient Chinese philosophy, such as yin - yang, five elements and the essence qi theory, has laid the foundation of the TCM theory; it specifies basic material and operation mode of the human life activity, and it also reflects the unity of human and external environment (including natural environment and social environment). TCM not only involves many natural science disciplines (such as astronomy, meteorology, geography, phenology, mathematics, etc.), but also pertains to social sciences and humanities. In order to establish the Top - level ontology of TCM, we should first draw the boundary, i.e. specifying the domains related to TCM, and state the relationships to other disciplines; secondly, the ontological research of the categories of yin yang, five elements and qi, etc. should be carried out; finally, various disciplines of nature, society and humanities should be taken into account additionally. Obviously, this is an arduous task which needs many experts from relevant fields to cooperate with each other and to finish it step by step [11].

### The essential difference between Chinese and Western thinking modes

Different from the western reasoning methods, influenced by positivism and empiricism, in the Chinese traditional culture the Chinese people comprehend the real world in a special way, that is to say, the Chinese traditional philosophies are generally determined by the idea of “harmony between man and nature” and are based on holism. The ancient Chinese people conceive complicated things as a whole or as a system, the parts of which are connected with each other, and mutually influenced. Above all, they pay their attention to analyzing the mutual relations between things. In contrast, the Western people tend to divide complex things into elementary units and then to find laws for this elementary level from which basic laws for the behavior of the complex system are deduced by means of logical methods. Hence, the Western thought exhibits a logical and reductive approach [8, 12]. The ancient Chinese traditional philosophy treats the origin of matter differently from the western tradition perspective: instead of looking for the material elements that compose the world inward, it studies the dynamic behaviors and behaviors’ function that the material world expresses outward, that is, the so-called “象” (the meaning corresponds to “image” or “phenomenon” in English) [13]. Therefore, the unique analogy, holistic thinking, the treatment according to syndrome differentiation of TCM, etc. result from this mode of thinking [13]. Hence, it is a big challenge to use traditional formal logic languages and ontological principles to express and capture adequately the unique thinking mode and perspective of TCM.

### Difficulties in practice

There are a lot of ambiguity and fuzziness in the semantic specification of concepts and terms in the TCM field. For example, the meaning of the term “Glycyrrhiza uralensis” (“甘草” in Chinese), which may refer to Glycyrrhiza uralensis of medicinal materials or Glycyrrhiza uralensis of decoction pieces, depends on context. This situation leads to difficulties in the standardization of terms. A balance between the ambiguity of a description in natural language and the certainty of a description in a formal language is needed. Domain experts are required to accurately understand knowledge and precisely capture the intension and extension of concepts [14]. In terms of conceptual system construction, the conceptual system of TCM’s basic theory is composed of yin and yang, five elements, viscera, meridians, qi and blood fluid, constitution, etiology, health preservation, prevention, treatment and five transport six qi, the part that has a relatively independent conceptual system and relationships between each other. A complete and uniform and clear hierarchical structure has not yet been established [15]. Compared with specific domain ontologies, concepts of TCM top- level ontology are much more abstract. The intension and extension of concepts, such as Qi, Yin and Yang, have not been reached a consensus. Therefore, the construction of a TCM top- level ontology will face more difficulties and problems.

#### Outline of the traditional Chinese medicine language system- semantic network (TCMLS-SN)

The traditional Chinese Medicine Language System- Semantic Network(TCMLS-SN) which is the backbone of TCMLS, specifies the relationships between concepts in TCMLS and provides an organization structure for all concepts in the basic thesaurus of TCMLS. TCMLS- SN conforms to the structural characteristics of the TCM theory and meets the digitization demand of TCM. Furthermore, the TCMLS- SN also provides links to related disciplines, such as medicine, biology, pharmaceutical technology, etc. The semantic network provides a kind of directory organization structure for all concepts appearing in the basic thesaurus of Chinese medical language system by means of semantic types. TCMLS- SN consists of 96 semantic types and 58 semantic relationships. It was re-designed, based on the semantic types of the UMLS and focused on introducing TCM concepts. There are two top categories of semantic types of TCMLS- SN: Entity and Event. Entity includes 40 semantic types, and Event includes 56 semantic types. These semantic types form a strict hierarchy and provide at least one semantic type to each concept [16]. The semantic relationships of TCMLS are based on the UMLS. There are two categories of relationships: the hierarchical is-a relationship, and non- hierarchical relationships, subsumed by the “associated- with” relationship. The hierarchical relationship is used to construct the TCMLS conceptual hierarchy, while non- hierarchical relationship is used to construct the network structure of TCMLS. The non- hierarchical relationships can be divided into five types: Physically related to, functionally related to, temporally related to, spatially related to and conceptually related to. Besides relationships, adopted from UMLS, TCMLS introduces several TCM specific relationships. For example, “Inter_exterior and _interior with is used to express the internal- external relationship between the viscera and the meridians”; “meridian entry”(归经) is used to express the function of Chinese medicinal belonging to meridians; “Opening at(开窍于)” is used to express a special relationship between the five viscera and the five officials according to the TCM theory.

Although TCMLS- SN has been verified by the use of Chinese medicine language system for nearly 20 years, as well as supported by the International Organization for Standardization (ISO), there are still some problems found during the practical applications. First of all, 100,000 concepts of TCMLS display an unbalanced distribution on the hierarchy structure. Some semantic types like “prescription,” and “Chinese Medicinal” contain a large number of child concepts, while some more specific semantic types such as “five elements (五 行),” or abstract semantic types such as “function of blood” contain only a few child concepts [11]. The need for achieving a structural balance requires further study. Secondly, according to the evaluation on the accuracy of semantic association of TCMLS carried by Jia [5], using TCMLS- SN to search the document database and check the documents in which concepts co- appear, the semantic relationships of TCMLS cover only 2% of actual usage. The cause of the low recall rate may include imperfect design of semantic relationships, as well as errors occurring in manual entries. In summary, TCMLS- SN, as the top- level frame of TCM, still has some problems in applications and, hence, further research is needed [17–19].

#### General formal ontology

General formal ontology (abbr:GFO), founded by the research group Onto-Med (Ontologies in Medicine) at the University of Leipzig, is an Upper Ontology, integrating Object and Process which is referenced as a basis framework for constructing domain ontologies related to various fields GFO is a component of ISFO (Integrated System of Foundational Ontologies), and ISFO is a part of an Integrated Framework for the Development and Application of Ontologies(IFDAO).Besides ISFO the system IFDAO includes the subsequently developed modules: a Library of Ontology Languages, and a System of Development Tools. This system of tools supports the development of domain oriented and generic ontologies GFO (General Formal Ontology) [20–24].

### Basic concepts and architecture

GFO aims at providing a foundational framework for constructing domain ontologies that could be derived from various fields. GFO has a three-layered meta-ontological architecture comprised of (1) an abstract top-level including set and item as the only meta-meta-categories, (2) a meta-level, called abstract core level containing meta-categories over the basic level, for example the meta-category of all categories. The entities of the world- being represented on the ATO-level by items – are exhaustively divided into categories and individuals, and individuals instantiate categories. Moreover, among individual objects and attributives are distinguished. (3) a basic level consisting of all relevant GFO-categories and relations. All basic relations and categories are presented as set-theoretical relations and set-theoretical predicates. Categories which are not contained within the basic level are called domain categories. Domain categories are related to a certain domain D of the world, and on the domain level they are not presented and considered as sets, but as entities of its own. Domain categories may be linked in a simple way to the basic level predicates of GFO, using domain upper linking axioms.

### Characteristics of GFO

According to [23] the distinguishing features of GFO may be summarized as following: It includes objects (3D objects) as well as processes (4D entities) and both are integrated into one coherent framework; GFO presents a multi-categorial approach by admitting universals, concepts, and symbol structures and their interrelations; it includes levels of reality; it is designed to support interoperability by principles of ontological mapping and reduction; it is presented by a set of formal axioms which might be added by metalogical links to domain specific ontologies; GFO is intended to be the basis for a novel theory of ontological modelling which combines declarative specifications with algorithmic procedures; it contains several novel ontological modules, in particular, a module for functions and a module for roles; and GFO is designed for applications, firstly in medical, biological, and biomedical areas, but also in the fields of economics and sociology.

### Compatibility with TCM

GFO holds a realist view, called Integrative Realism, at the world and assume the existence of a real world which is independent from the subject. This kind of realism postulates a particular relation between the mind and the independent material reality. This relation connects dispositions of a certain type, inhering in the entities of material reality, with the manifold of subjective phenomena occurring in the mind. This relation can be understood as unfolding the real world disposition X in the mind’s medium Y, resulting in the phenomenon Z. see [24]. By means of the analysis and reconstruction of principles of Yin-Yang within GFO, an unique Yin-Yang dialectic ontology is elaborated and presented in [25]. Furthermore., we yield an extension of GFO, called GFO*, that could include some kind of new categories derived from East culture, especially from Chinese traditional culture, for example: yin yang, Qi, meridian. Generally, GFO aims to provide a common foundational framework across various knowledge domains which may result from different cultural contexts, and makes sense of a mutual understanding and communication between the western- and eastern- culture from different standpoints; furthermore, it provides a bridge to exchange philosophical ideas and to integrate medicinal knowledges between western medicine and traditional Chinese medicine (TCM). In most cases, as we are to construct some domain ontologies related to TCM, we often have to consider of how to analyze or how to describe some abstract- or particular- entities; these entities may involve categories or individuals in terms of GFO; such as: Qi, Yin Yang, Zheng(证), meridians, the processing of herbal medicine, the diagnose in TCM etc. Above all, we must have a deep understanding about these entities. When analyzing these entities at the ontological level, we find out that many of them are involved in various categories in GFO. For example: the concept Qi can be understood as the most fundamental matter, but in some context also as a functional process or as a property. On the other hand, the upper ontology, upon which our domain ontologies are built, must provide a complete classification and a rational model to identify and to cover all these entities. In a sense, GFO can be understood as an ontology about “processes”, or rather to say “object-process integration”. In GFO, processes are the most fundamental category of individuals. First, it distinguishes two categories in GFO: objects (also called continuants) and presentials. Objects persist through time; they are reflected by universals, called persistants, which present the object’s identity over time. An object Obj exhibits at any time-point t of its life-time an entity Obj(t), called presential, being wholly present at that time-point t. Presentials are dependent entities, they depend on objects and on the processes, which are – according to the integration law – associated to objects. Second, in GFO, Temporal complexes (or processual structures) are the most general kind of concrete individuals which have a temporal extension. The temporal extension of a temporal complex is a mereological sum of a non -empty set of chronoids. Processes form the most important sub-class of temporal complexes, and occurrents centers around the notion of process. Occurrents are dependent entities that are related to processes in various ways. Furthermore, in GFO they are classified into Change, Event, History, Action, continuous process, and discrete process, respectively [23].

## Methods

We start with analyzing the technical specification based on TCMLS, considering some obvious classification errors and problems of ambiguity of semantic expression in TCMLS-SN, followed with using a top-down approach for building a middle level ontology which based on the framework General Formal Ontology (GFO), take account of compatibility with TCM related concepts, turn out the results of a modification of the current TCMLS-SN, called GFO-TCM.

### GFO-TCM: A Core ontology for the TCMLS

**Fig. 1:**
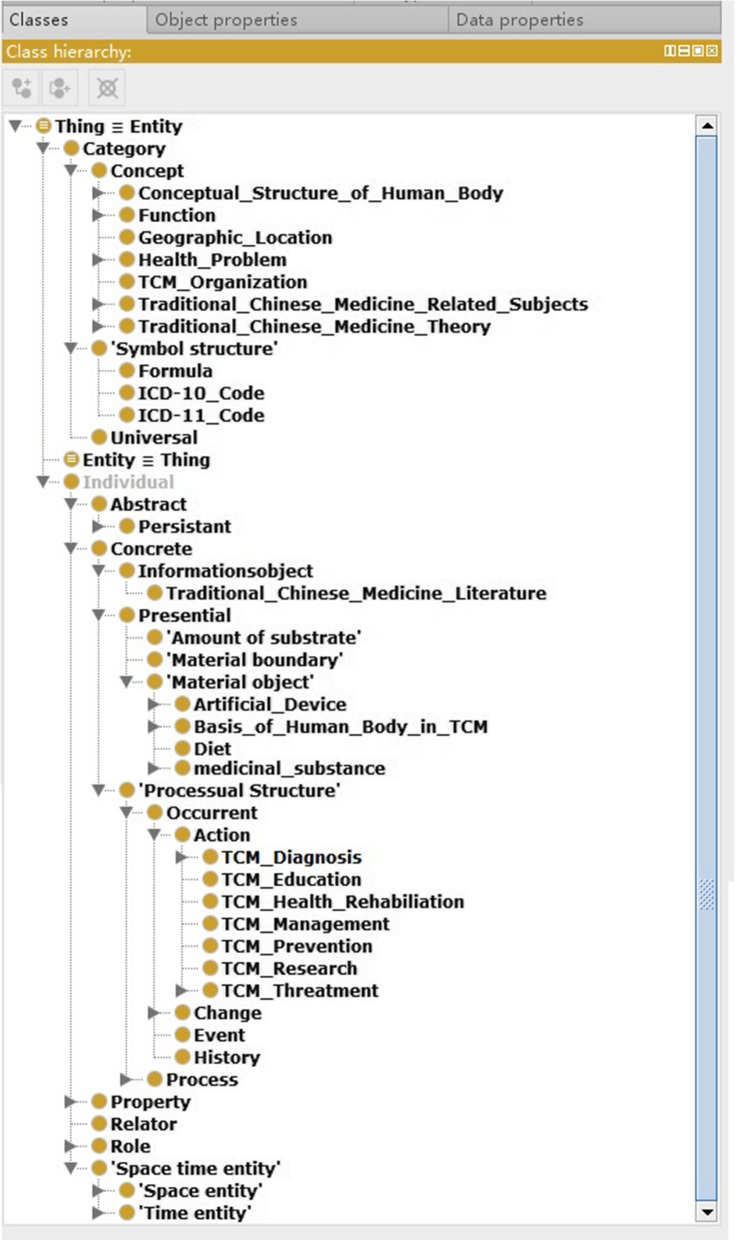
Basic structure of GFO-TCM

In this section, we introduce a core domain ontology based on GFO, called GFO-TCM. Although GFO-TCM directly arises from TCMLS-SN, it adopts a new conceptual classification and rebuilds semantic relations of the framework GFO which differs from the way of TCMLS-SN. We analyzed the taxonomy and semantic relations of TCMLS-SN with respect to entailment and coherence, reconstructed the semantic type and relations according to the standpoints of GFO. To point at the phenomenon of vagueness of concepts and of data redundancy, as well as the defect of taxonomy according to the specification in TCMLS-SN, the GFO-TCM aims at providing a more reasonable solution with a unified semantic criterion that can be continuously improved. Our work is oriented to the specific applications depending on demands, that is to say, an analog approach as presented in [26], should be further elaborated for TCM fields, i.e. for information retrieval, information extraction, text mining, as well as for clinical decision support systems. The basic structure of GFO-TCM shown as Fig. 1.

#### Semantic types

GFO-TCM can be understood as a domain core ontology. According to [23, 24]. the entities in GFO-TCM are also divided into *Categories* and Individuals*,* whereas categories are entities that are expressed by predicative terms of a (formal or natural) language and that can be predicated of other entities, and individuals instantiate categories.

In GFO categories are further classified into *concepts*, *universals,* and *symbol structures*. The definitions and the mutual relations between them can be understood as follows: *Concepts* are categories that are represented as meanings in someone’s mind. Concepts are a result of common intentionality which is based on communication and society [27]. We hold that *universals* can only be accessed through concepts, hence for the establishing of knowledge the category of concepts is the most important one. *Symbol structures* are signs or texts that can be instantiated by tokens.

Individuals are entities that are not instantiable, they are divided into space-time entities, concrete and abstract individuals, and in properties, relators, roles, and so on [23, 24].

Generally speaking, in the process of constructing this core domain ontology, called GFO-TCM, the semantic types and relations in TCMLS-SN have been mapped and classified into a meta -level with link axioms, namely, into the abstract core ontology (ACO) of GFO, as sub classes of categories and individuals respective, which is described below:

### GFO: Categories-concepts

In TCMLS-SN, meridian and collateral, acupuncture points are classified into the semantic types of “Basic material in the human body in Chinese medicine [10]. According to the traditional theory of Chinese medicine, the meridian system consists of meridians (经脉) and collaterals (络脉), meridians and collaterals are pathways in which the qi and blood circulate and through which the viscera and limbs are connected, allowing the upper- lower- and interior- exterior portions of the body to communicate,” [28]. Acupuncture points are parts of the meridian system, located on the body’s surface, which can be transfused with the qi and blood [29]. According to the viewpoints of TCM, the doctrine of meridians and acupoints is the result of experience of many medical practices, done by ancient people, which has a long history, and which includes, among others, acupuncture, tuina (TCM Massage), and Qigong [30, 31]. Although many hypotheses in relation to their essential properties have been proposed, some investigation suggested: there is no anatomical and physiological basis or evidence for the concepts such as meridians and acupuncture points [32, 33]. For this reason, we hold that these concepts should be classified into a new type, say “*conceptual structure of human body”*. However, considering the spatial axioms of boundaries of material structures in GFO, meridians and acupuncture points must satisfy these axioms, i.e. they occupy some three-dimensional space regions, and possess material boundaries in the human body.

In GFO-TCM, the Health Problems differ from the semantic type of “etiology pathogenesis and disease TCM”; it seems that the meaning of this term is inane or vague, and can result in some confusions, because of the viewpoint of GFO that the entities, i.e. Etiology, Pathogenesis and Disease, can be ascribed to different categories. Hence, we intend to introduce some different hypernyms in the GFO-TCM, according to [34]. The health disorders and the health care related issues should be statistically analyzed and classified into the International Statistical Classification of Diseases and Related Health Problems. (abbr. ICD). Therefore, the semantic types: Diseases, Symptom of TCMLS-SN have been ascribed into Health problems of GFO-Concepts. On the other hand, in the next version of ICD-11 (Mortality and Morbidity Statistics) that is to be published in 2018 [35], a new module of the Traditional Medicine conditions -Module I - have been introduced. This class is further divided into Traditional medicine disorders (TM1) and Traditional medicine patterns (TM1), to distinguish them from the term of disease in western medicine; we ascribe them also into the *GFO-Health Problems* in GFO-TCM. But for TCM Etiology and TCM Pathogenesis, we hold that they should be ascribed into the class of the theory of TCM, because of they are usually considered as theories in TCM.

### GFO: Categories-symbol structure

In TCMLS, a formula(方剂) is considered as a kind of artificial material, but this approach is questionable, because formulas may denote other entities, for examples, some components of herbal drugs. However, formulas, as conceptual entities, themselves are not natural material structures, actually formulas, in GFO, are rather considered as some kind of symbolic structures with semantic content, but not as a particular artificial material. We assume that formulas are considered as symbol-structures, which are equipped with some definition. The instances of this symbolic structure, being tokens, denote some particular object. Let us consider an example of a formula, say, bupleurum decoction (柴胡汤), consisting of various herbal drugs. Assume a doctor A is writing the bupleurum decoction as a formula on paper for his patient B; the patient B must firstly recognize and classify this perceived textual token, grasped from a paper or as vocal token, from his doctor’s A mouth. Secondly, B must put these tokens into some equivalent class, then there emerges some concept about bupleurum decoction in B’s mind which is exemplified by some particular individuals, such as the decoction’s components and the usage if this drug. Generally speaking, this is an information object with respect to a ternary relation, i.e. we assume, an information object is represented by a triple that is composed of three different components, a symbol, a token and an object which is denoted by this token.

**Fig. 2:**
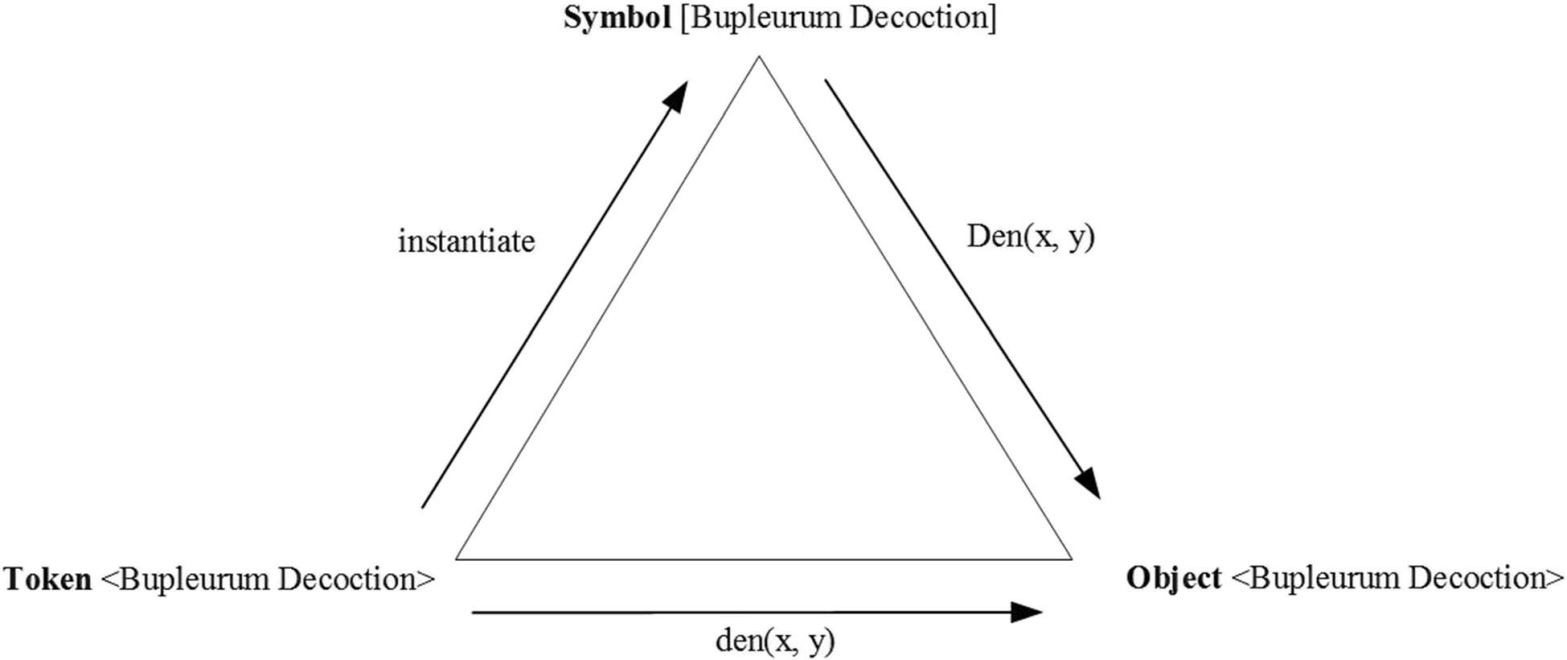
The triple of an information object

As Fig. 2 shown below. *IO:=(Symbol, Token, Object).*

Furthermore, we propose that the related binary relations among this Triple can be expressed as following axiom:

$$ \mathrm{Den}\left(\mathrm{S},\mathrm{Obj}\right)\ \mathrm{iff}.\exists \mathrm{t}\ \left(\mathrm{t}::\mathrm{S}\wedge \mathrm{den}\ \left(\mathrm{t},\mathrm{Obj}\right)\right) $$

In TCMLS-SN, Traditional Chinese Medicine Literature is a subclass of the semantic type of conceptual entities. We hold that - apart from literature -, there are numerous other TCM-related data resources, for example data, presented in form of pictures, videos, cassettes, DVD, or of some other medium, which may contain some valuable information on TCM research as well. Inspired by the thoughts of [36, 37], we propose a new semantic Type: the GFO-Information object, that is considered as an upper concept for the literature. This Information object can be represented as a triple that already mentioned in above.

### GFO: Inividual-processual structure

According to [23] Processual Structure (or temporal complexes) are the most general kind of concrete individuals which have a temporal extension. It can be further divided into *Occurrent* and *Process*. The occurrents are further classified into actions, events, changes, and histories respective. Actions are occurrents which are caused by some presential (the agent) at every (inner and outer) time-boundary of the chronoid framing the occurrent. According to this viewpoint we classify the TCM -diagnosis, −education, −health rehabilitation, −management, −prevention, −research, −treatment into the GFO-occurrent-actions; and the pharmaceutical procedure into the GFO-occurrent-process. On the other hand, in GFO, a change in the technical sense refers to a pair of process boundaries. Either at coinciding boundaries (then it comes close to notions like “punctual” or “instantaneous event” as well as “moment” - in a temporal reading), or at boundaries at the opposite ends of a process of arbitrary extension. According to this standpoint we ascribe two new categories, named change in five phases(五行转换), and change in yin yang(阴阳变化)into the sub class of the GFO-change. However, we hold that the principle of the two is very important for the TCM-related research, therefore, they should be systematically investigated and analyzed at an ontological level, but is not yet included in the context of this paper.

### GFO-property

In GFO, a property is considered as a specific entity that is a dependent entity which another entity, the bearer, can possess. In TCMLS-SN, Constitution(体质) is ascribed into the semantic type of “Basic material in the human body in Chinese medicine” of Physical and presentational object. However, according its definition given in [10]: it is the characteristics of an individual, including structural and functional characteristics, temperament, adaptability to environmental changes and susceptibility to disease. It is relatively stable, being in part, genetically determined and in part, acquired. Hence, we hold that the assumption of the constitution of human body in the theory of TCM should be classified as a subclass of GFO-Property. For the same reason, the nature of Chinese medicinals of TCMLS-SN can also be ascribed into the GFO-property as well.

#### Semantic relations

Basically, in GFO-TCM we inherit the basic relations of GFO and adopt the twelves special relations of TCMLS-SN,. i.e. inter-exterior and -interior with (相表里), opening at(开窍于), inhibit (相恶), antagonize(相反), suppress(相畏), mutual reinforce(相须), assist(相使), restrain(相杀), engendering(生), restraining(克), overwhelming(乘), rebellion(侮). Using this approach, we hold that the GFO-TCM maintains the coherence with GFO and, at the same time, keeps the compatibility with TCMLS to a certain extent. Besides this, we introduced a new relation, named hasCompatibility (配伍) as an upper relation, subsuming the relations antagonize, assist, inhibit, mutual reinforce, restrain, suppress, respective.

## Results

Through comparison with TCMLS-SN, according to viewpoints of GFO, some semantic types and relations were reconstructed within GFO-TCM. We propose a middle level ontology for TCMLS which may support entailment and ensures coherence. Furthermore, we establish a mapping which provides a more reasonable framework that is based on a unified semantic criterion. This framework is directed at application scenarios and can be further updated, refined and extended.

### Comparison with TCMLS-SN

The semantic types for the field of Chinese medicine should be chosen with respect to pragmatism and integrity, that is, the coverage and frequency of its use in reality should be considered. Hence, for domain modelling we focus on those semantic types that are commonly used in TCMLS. For example: Formulas, Chinese medicine literature, Chinese medicine, medical personages, animals and plants, diagnostic processes, diseases or syndromes, chemical components of Chinese medicine, diet, Chinese medicine institutions, etc. On the other hand, GFO-TCM also continues to retain some of the abstract upper semantic types that are not commonly used in TCMLS, but have the nature of top-level classification nodes: for example, TCM theory, TCM related subjects (or disciplines), TCM research, etc.. Additional new Semantic types, such as the conceptual structure of the human body, information objects, health problems, five-phase changes, yin-yang changes, etc. are added.. GFO-TCM is intended to be a domain core ontology of TCM. Based on this, there is the need to continuously update and/or to construct new domain ontologies of TCM in the future; these ontologies must possess appropriate abstractness and sufficient compatibility. Therefore, we have abandoned some entities that are excessively abstract or generalized, because these concepts are quite vague and ambiguous, such as the *etiology and pathogenesis and disease of TCM*, *property and function of Chinese medicine*,etc.Some semantic types that cannot be immediately used in TCMLS, such as meridian entry, acupuncture prescriptions, functions of traditional Chinese medicines, functions of formulas, etc. have been preliminarily abandoned or merged to a certain extent. Furthermore, those semantic types which are misclassified, for example, psychological function, Chinese medicine psychology, mind, emotions, etc. also have been preliminarily deleted or merged. The integration of these notions into GFO-TCM needs further research; GFO provides four ontological regions: the ideal entities, the material objects, the psychological entities, and the socio-systemic entities. These different regions play a decisive role in the further development of GFO-TCM. In the meantime, a new semantic type: ICD-Code is introduced into the GFO-TCM, as a sub-category of the GFO-Symbol Structure, according to [35]. We expect that in the future some entities should be referred to this category of ICD-Codes if necessary.

### Mapping

In the previous sections we described the basic architecture (or entities) of GFO-TCM. In fact, except for a few abstract upper nodes, most of the semantic types from TCML-SN can be mapped into the GFO-framework. However, for the reasons mentioned above, the two are constructed according to different classification principles, namely, the GFO-TCM is based on the basic framework of the GFO, and the TCMLS-SN is established that referred to the conceptual system of UMLS-SN, therefore, the two cannot carry out an exact and consistent matching or mapping. For example, formulas are regarded as an artificial substance in TCMLS-SN, but in GFO-TCM considered as a symbolic structure, which can refer to some information objects; in TCMLS-SN, Constitution is considered as a basic material of the human body, by comparison in GFO-TCM as a property with respect to the criterion of constitution classification of human bodies; additionally, in TCMLS-SN meridians and acupressure points are assigned to the semantic type of the basic material in the human body in Chinese medicine, but in GFO-TCM it is considered to be a conceptual structure of human body. The compatibility of Chinese medicine (or Chinese medicinal combination 配伍) in TCMLS-SN regarded as a traditional Chinese medicine function, but considered as an object property in GFO-TCM. The Table 1 shows a rough mapping among them.

**Table 1:** The mapping between GFO-TCM and TCMLS-SN

GFO-TCM	TCMLS-SN
Entity	Thing
Category	-
Concept ∪ Symbol ∪ Universal	Conceptual Entity
Conceptual Structure of Human Body	-
Function	Phenomenon and Process
	Physiology Phenomenon or Process
	-TCM Physiological Function
Health Problem (-Disease,-Symptom, -Traditional Medicine Conditions)	Phenomenon And Process
	-Etiology Pathogenesis and Disease TCM
TCM Related Subjects	Related Subjects of TCM ∪ TCM Subjects
TCM Theories	Traditional Chinese Medicine Theory
Symbolic Structure-Formula	Medical Substance-Formula
Individual	Physical And Presentational Object ∪
	Event
Abstract-Persistant	-
Concrete	Physical And Presentational Object
Presential-Material Object	
Informationsobject	-
Processual Structure	Event
Occurrent-Action ∪	Activity
Process-Pharmaceutical Procedure	
-Change	-
Property	Property and Function of Chinese Medicinal
-Nature of Chinese Medicinal	-Nature of Chinese Medicinal
-ObjectProperty: hasCompatibility	-Function of Chinese Medicinal
	Chinese Medicinal Combination

## Related applications

GFO-TCM is a core ontology based on GFO’s framework, it is intended to lay the foundation for the large-scale construction of a TCM knowledge base with respect to various knowledge resources in the field of traditional Chinese medicine. These knowledge resources can provide relevant explicit Knowledge in terms of applications and services for the users. Furthermore, GFO-TCM is proposed to be a domain core Ontology, i.e. considering GFO-TCM as framework, a navigation system of TCM knowledge graph should be established to visually represent the specific concepts and interrelated knowledge nodes of TCM. In the meantime, a user can quickly retrieve the required information content according to his interest. The relevant results are intuitively represented in the form of a knowledge card, that is, knowledge and concepts are related to the retrieved content. Ultimately, based on the core ontology GFO-TCM of the Chinese medicine, the goal is to build a Wikipedia-style knowledge base system for Chinese medicine, which can support various related specific applications, such as for providing a navigation of Chinese medicine knowledge in terms of the related concepts. That means, according to different search items a quick semantic retrieval and information extraction should be made available for users, by way of efficient and accurate knowledge services, and for the development of expert decision support systems as well, etc.

## Discussion

In summary, we proposed the first version of TCM core ontology, based on GFO’s framework, which is directly derived from TCMLS-SN. Our paper and the future work focusses on issues discussed in chapter 3. Furthermore, we are planning, to realize some specific application related scenarios. According to [24], a domain is determined by classification principles that are to be used for the construction of concepts and a set of views assumed which determine the considered objects of domain. In view of the close relationship between traditional Chinese medicine and Chinese traditional culture, this relationship should be deeply analyzed and elaborated in the framework of GFO. But also, the relationship to other disciplines must be investigated. Finally, some basic categories of TCM, such as Yin Yang, Qi, Five Elements, etc. must be further analyzed with respect to the exploration of how to understand and reconstruct these categories at an ontological level. Existing domain ontologies, established in the field of TCM, should be further reviewed, reconstructed, and then integrated into the framework of GFO. At present, there is a large number of mature and available ontologies in the field of biomedical sciences (for example, OBO Foundry). There are also some ontologies related to Chinese medicine that have been put into use. The problem is how to overcome the differences or how to reconcile the conflicts among these ontologies.

## Conclusions

The goal of our work is to construct a formal middle-level ontology that is compatible with both the traditional medical terminology system and modern medical terminology standards. It is intended to satisfy functional requirements which are relevant for natural language processing, information extraction, semantic retrieval, clinical decision support in the field of traditional Chinese medicine. It also provides a foundation and methodology for building a large-scale, unified semantic and extensible knowledge graph platform. In the future, we intend to implement some specific applications derived from this paper, some technologies or tools which developed by the IMISE team, such as expert2owl tool [38] or GFO-search ontology [26] are already available. Hence, the goal aims at providing an (semi-) automatic tool supporting the manual approach to build ontologies for the TCM experts. Another important topic is basic research in the field of Chinese medicine with respect to information extraction, and data mining from the TCM related big data as well as the building of TCM clinical decision support systems on demand.

## Abbreviations

GFO: General Formal Ontology
TCM: Traditional Chinese Medicine
TCMLS-SN: traditional Chinese Medicine Language System- Semantic Network
UMLS: Unified Medical Language System
